# Increased androgen receptor expression in serous carcinoma of the ovary is associated with an improved survival

**DOI:** 10.1186/1757-2215-3-14

**Published:** 2010-06-17

**Authors:** Björn Nodin, Nooreldin Zendehrokh, Jenny Brändstedt, Elise Nilsson, Jonas Manjer, Donal J Brennan, Karin Jirström

**Affiliations:** 1 Center for Molecular Pathology, Department of Laboratory Medicine, Lund University, and Skåne Regional Laboratories, Malmö, Sweden; 2 Department of Clinical Sciences, Division of Surgery, Lund University, Skåne University Hospital, Malmö, Sweden; 3 The Malmö Diet and Cancer Study, Skåne University Hospital, Malmö, Sweden; 4 UCD School of Biomolecular and Biomedical Science, UCD Conway Institute, University College Dublin, Dublin, Ireland

## Abstract

**Background:**

Altered androgen hormone homeostasis and androgen receptor (AR) activity have been implicated in ovarian carcinogenesis but the relationship between AR expression in ovarian cancer and clinical outcome remains unclear.

**Methods:**

In this study, the prognostic impact of AR expression was investigated using immunohistochemistry in tissue microarrays from 154 incident cases of epithelial ovarian cancer (EOC) in the prospective, population-based cohorts Malmö Diet and Cancer Study and Malmö Preventive Project. A subset of corresponding fallopian tubes (n = 36) with no histopathological evidence of disease was also analysed.

**Results:**

While abundantly expressed in the majority of fallopian tubes with more than 75% positive nuclei in 16/36 (44%) cases, AR was absent in 108/154 (70%) of EOC cases. AR expression was not related to prognosis in the entire cohort, but in the serous subtype (n = 90), AR positivity (> 10% positive nuclei) was associated with a prolonged disease specific survival in univariate (HR= 0.49; 95% CI 0.25-0.96; p= 0.038) and multivariate (HR= 0.46; 95% CI 0.22-0.97; p= 0.042) analysis, adjusted for age, grade and clinical stage.

**Conclusions:**

AR expression is considerably reduced in EOC as compared to fallopian tubes, and in EOC of the serous subtype, high AR expression is a favourable prognostic factor. These results indicate that assessment of AR expression might be of value for treatment stratification of EOC patients with serous ovarian carcinoma.

## Background

Epithelial ovarian carcinoma (EOC) is the second most common and the most lethal malignancy of the female reproductive tract [1]. Etiological factors involved in ovarian carcinogenesis remain poorly defined, and effective treatment protocols are limited. Alterations in androgens and androgen receptor homeostasis have been implicated in ovarian carcinogenesis and progression [2-5].

While several immunohistochemistry (IHC)-based studies have confirmed widespread AR expression in EOC [6-8], data describing it as a prognostic biomarker are relatively sparse. One study describing a large series of tumors (n = 322), found no association between AR protein expression and clinical outcome [8], however individual histological subtypes were not examined. Increased levels of AR mRNA have been described in cells from normal ovarian surface epithelium as compared to ovarian cancer cells, the majority of which were derived from serous tumors [9]. We are, however, unaware of any studies describing AR expression in fallopian tubes, from which a substantial but not yet not fully appreciated proportion of serous ovarian carcinomas are thought to arise [10].

The purpose of this study was to analyze the prognostic impact of AR expression in 154 EOCs collected from two population-based, prospective cohorts. Based on the *in vitro *data described above [9], our hypothesis was that AR protein expression may be down-regulated in EOC compared to fallopian tubes and the prognostic value of AR would become more obvious when tumors were stratified into serous and non-serous histological subtypes.

## Methods

### Patients

Tumors (n = 154) from all incident cases of invasive EOC that had occurred in two prospective, population-based cohorts, the Malmö Diet and Cancer Study (MDCS)[11] and Malmö Preventive Project (MPP) cohorts [12] up to Dec 31^st ^2007 were collected and histopathologically re-evaluated. The MDCS was initiated in 1991 and enrolled 17035 healthy women [11]. The MPP was established in 1974 for screening with regard to cardiovascular risk factors and enrolled 10.902 women[12].

The standard surgical management was a total abdominal hysterectomy, bilateral salpingo-oophorectomy and omentectomy with cytological evaluation of peritoneal fluid or washings. Routine pelivic lymphadenectomy was not performed. Residual disease was resected to less than 1 cm where possible. Volume of residual disease was not availabe. Standard adjuvant therapy was combination of paclitaxel and platinum-based chemotherapy.

Median age at diagnosis was 62 (range 47-83). Information on cause of death was obtained by matching with the Swedish Cause-of-Death Registry. After a median follow-up of 2.67 years (Range 0-21.14 years) 105 patients were dead, 98 from ovarian cancer. Approval was obtained from the Ethics committee at Lund University (Ref no 335-08) Study design, methodological and technical considerations, as well as data presentation were based on the REMARK criteria [13]

### Tissue microarrays and immunohistochemistry

TMAs were constructed as previously described[14]. Two 1.0 mm cores were taken from viable, non-necrotic tumor areas, when possible from both ovaries, and from concomitant peritoneal metastases (n = 33). Fallopian tubes with no evidence of histological disease were also sampled from 38 cases.

Four μm TMA-sections and 3μm full-face sections were deparaffinised and rehydrated. Heat mediated antigen retrieval (pH = 9) was performed using the PT-link system and IHC was performed in the DAKO Autostainer system (Dako, Glostrup, Denmark) using mouse monoclonal anti-AR antibody (1:200 dilution; AR 441, LAB VISION, Warm Springs, CA), anti-ER antibody (1:50 dilution; M 7047 Dako), and anti-PR antibody (1:400 dilution; M 3569 Dako).

To control for heterogenous expression patterns, IHC was also performed on full-face sections from 15 randomly selected cases and compared to corresponding cores. AR expression was also examined on full-face sections from fallopian tubes obtained from 10 patients who had undergone hysterectomy for benign disease.

### Statistics

Spearman's Rho correlation and the χ^2^test were used to estimate the relationship between AR expression and clinicopathological parameters. Kappa-statistics were used as a measure of agreement between scoring of tissue cores and full-face sections. Kaplan-Meier analysis and log rank test were used to illustrate differences in ovarian cancer specific survival (OCSS) between strata. Cox regression proportional hazards models were used to estimate the relationship between survival and AR status, age, stage and grade. All calculations were performed using SPSS version 17.0 (SPSS Inc, Chicago, IL). P values < 0.05 were considered statistically significant.

## Results

### AR expression in fallopian tubes, primary and metastatic EOC

**Figure 1:**
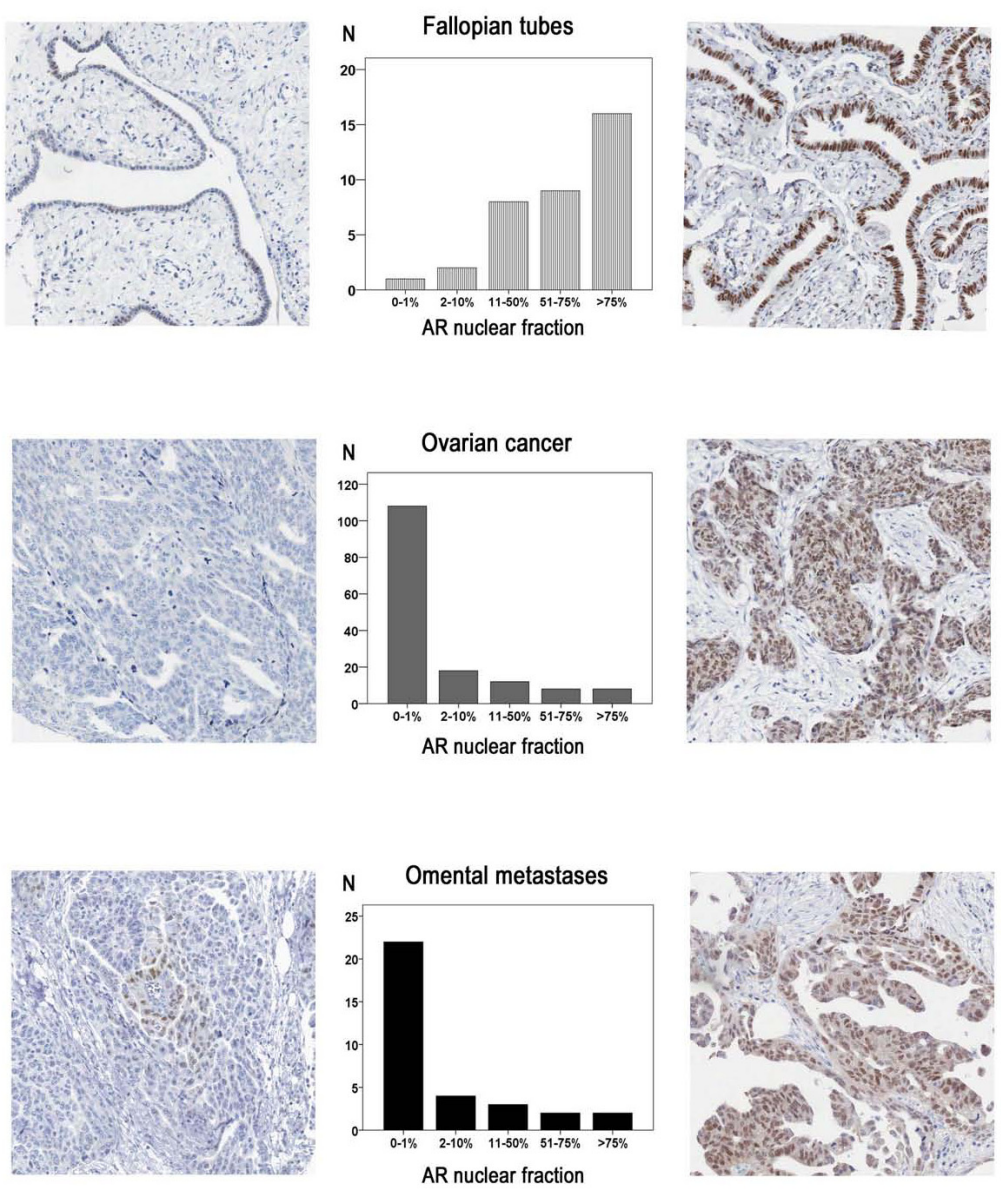
**Immunohistochemical AR staining and distribution in fallopian tubes, ovarian cancer and omental metastases**. AR nuclear staining was assessed as the percentage of positive tumor cells (grading 0-1%, 2-10%, 11-50%, 51-75%, >75%).Examples of tumors with low AR expression are visualized in the left panels and tumors with high expression in the right panels. Bars in the middle represent the distribution of positive cases in absolute numbers.

Thirty-six of the 38 fallopian tubes were suitable for analysis. AR protein expression was evident in the majority of fallopian tubes with > 75% positivity seen in 44% (n = 16) of cases (Figure 1). AR was also abundantly expressed (> 50%) in 10 fallopian tubes with a benign diagnosis (data not shown).

All primary tumors (n = 154) and metastatic deposits (n = 33) were suitable for analysis. Compared to tubal epithelium, AR protein expression was lower in primary tumors and metastases, with absent expression in 70% (n = 108) of primary tumors and 67% (n = 22) of metastatic deposits (Figure 1). AR expression in primary tumors correlated with expression in metastases (R= 0.95, p < 0.001) particularly when serous carcinomas (n = 90) were analyzed separately (R = 0.97, p < 0.001). No correlation was seen between tubal AR expression and expression in either primary or metastatic tumors. As samples from all three locations were only available for six patients, this study did not allow for a meaningful analysis of AR expression related to individual tumor progression.

AR expression in full-face sections correlated with TMA-based scoring (kappa-value 0.87, p = 0.001, n = 15), suggesting that AR is a suitable protein for TMA-based analysis.

### Correlation between AR expression and clinicopathological parameters

No significant association was evident between AR expression in primary tumors and conventional clinicopathological parameters in the entire cohort (n = 154) (Table 1). In primary tumors, AR expression was associated with ER and PR positivity (Table 1). Subset analysis of serous carcinoma's (n = 90), revealed that the association between AR and ER positivity remained significant, whereas the relationship with PR expression was lost (Table 1). AR expression was also associated with well-differentiated serous tumors (Table 1).

**Table 1:** Correlations between androgen receptor status and patient and tumour characteristics in all tumours and serous carcinomas respectively

	All tumours			Serous carcinoma		
				
	AR low	AR high		AR low	AR high	
	126	28	p-value ±	71	19	p-value ±

						
**Age**						
median(range)	62(47-83)	63(50-79)	0.800	63(47-83)	64(50-79)	0.514
						
**Histological subtype**						
Serous	71	19	0.207			
Endometroid	28	7				
Other	27	2				
						
**Stage**						
I	22	4	0.667	4	2	0.136
II	16	2		5	2	
III	57	18		41	13	
IV	19	3		13	1	
missing	12	1		8	1	
						
**Differentiation grade**						
High/intermediate	37	10	0.511	14	7	0.015
low	89	18		57	10	
						
**ER**						
Negative	62	5	0.003	28	2	0.024
Positive	60	21		40	15	
missing	4	2		3	2	
						
**PR**						
Negative	103	18	0.033	61	15	0.676
Positive	19	9		9	3	

AR = androgen receptor, ER= estrogen receptor, PR= progesterone receptor

< 10% positive nuclei used as cutoff for AR, ER and PR positivity

± Mann Whitney u-test for comparison of medians and Chi-square test for categorized variables

Cases with missing values were not included in the analysis

### AR expression in relation to survival

**Figure 2:**
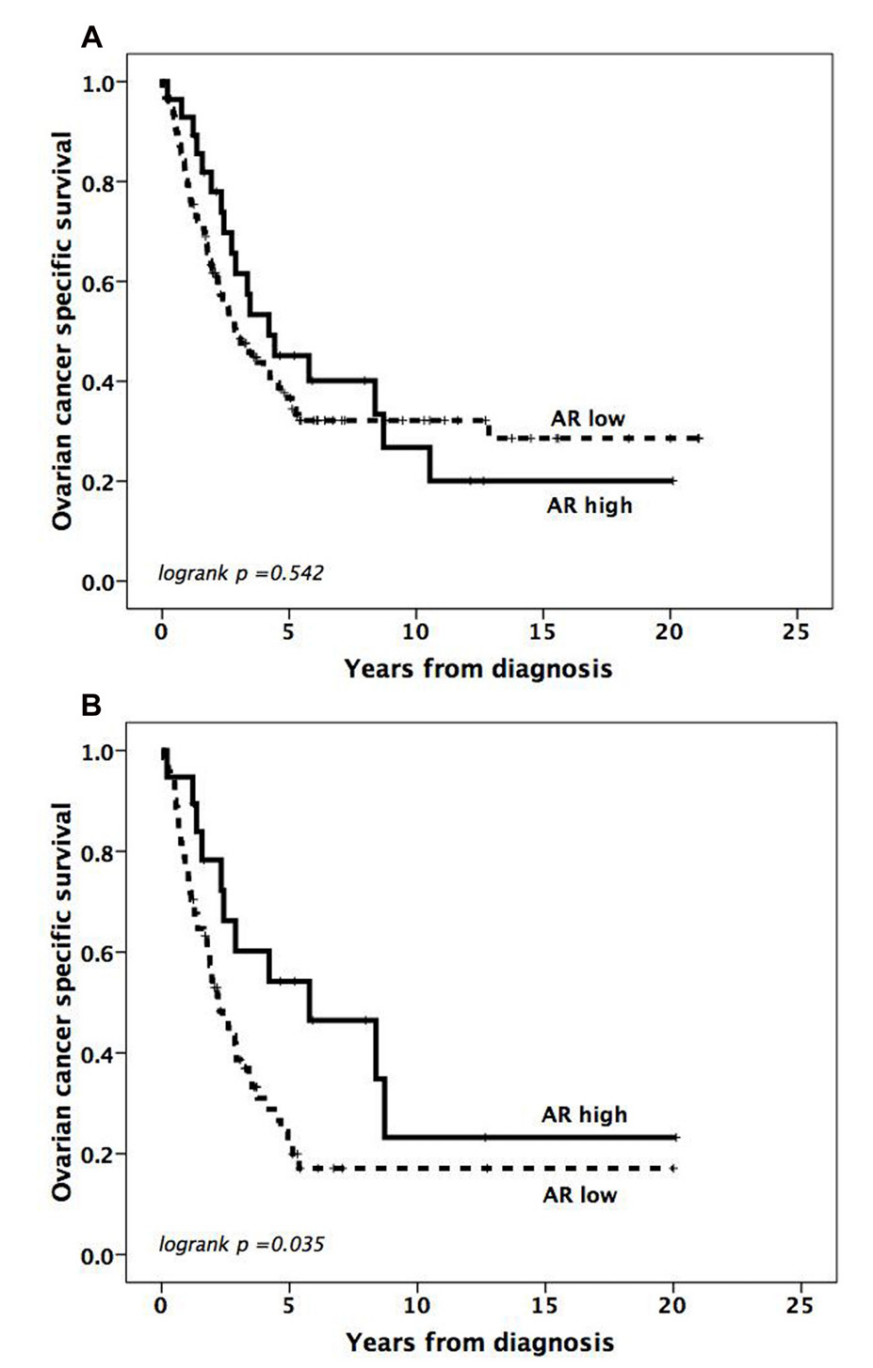
**Impact of androgen receptor expression on ovarian cancer specific survival**. Kaplan-Meier curves visualizing OCSS according to AR expression in all tumors (A) and serous carcinomas (B), using a threshold of 10% positive nuclei to define low and high AR expression. The total number of events was 52/71 (73%) in AR high serous tumors and 11/19 (58%) in AR low serous tumors.

Analysis of the entire cohort (n = 154) revealed no relationship between increased AR expression (> 10%) in primary tumors and outcome (Figure 2A). However, subset analysis in serous carcinomas (n = 90) revealed that increased AR expression was associated with a prolonged OCSS (p = 0.034) (Figure 2B). Cox univariate analysis confirmed the association between AR and OCSS in serous carcinomas (HR= 0.49; 95% CI 0.25-0.96; p= 0.038) and this association remained significant in a multivariate model controlling for age, grade and stage (HR= 0.46; 95% CI 0.22-0.97; p= 0.042). AR was not prognostic in non-serous carcinomas (data not shown).

## Discussion

Evaluation of AR protein expression in 154 EOC cases from two large, prospective population-based studies demonstrated frequent expression of AR in fallopian tube epithelium irrespective of the presence of ovarian cancer and decreased AR expression in primary ovarian tumors and metastatic deposits. While not conferring a prognostic value within the entire cohort, reduced AR expression was an independent predictor of decreased OCSS in serous tumors. The main limitation of this study is the absence of data on residual disease and future studies of AR expression in EOC should incorporate this in any multivariate analysis.

While associated with AR expression, neither ER nor PR expression correlated with survival in this study. Such findings contrast with Lee *et al*. who reported PR, but not ER or AR expression as an independent predictor of good prognosis [8]. In their study, however, the prognostic value of hormone receptors was not analysed in strata according to different histological subtypes, an approach that has been deemed an essential component of EOC biomarker studies[15]. These findings further highlight the heterogeneity of ovarian cancer, which should not be considered as a single disease, but rather several distinct entities with different clinical behaviours. These entities are in part reflected in histopathological characteristics and therefore, to obtain better prognostic and predictive information biomarkers should not only be assessed across entire cohorts, but also in histological subgroups.

Although androgen receptors are expressed in normal ovarian surface epithelium[16], we are not aware of any previous reports describing AR expression in tubal epithelium. Recent reports have suggested that a significant proportion of serous carcinomas arise within the fimbrial tubal epithelium [10,17,18]. Our findings indicate that malignant transformation could involve a downregulation of AR in certain EOC cases. AR expression in primary ovarian tumors and metastases was quite similar, suggesting that downregulation of AR occurs early in ovarian carcinogenesis.

This is to our knowledge the first report on AR expression in EOC from population-based cohorts, potentially representing a selected part of the background population. Nevertheless, as established prognostic parameters, i.e. clinical stage and histological grade, are highly significant indicators of survival in this cohort, its use for assessment of investigative prognostic markers is justified.

## Conclusions

These data demonstrate that AR is an independent marker of prolonged OCSS in patients with serous carcinoma of the ovary, and thus a potentially relevant biomarker for treatment stratification in this subgroup. Our findings also highlight the need for further studies investigating the influence of both lifestyle-related and genetic factors in relation to ovarian cancer risk in general and to AR-defined subtypes in particular.

## Abbreviations

AR: Androgen Receptor; EOC: Epithelial Ovarian Cancer; OCSS: Ovarian Cancer Specific Survival; TMA: Tissue Microarray; IHC: Immunohistochemistry; ER: Estrogen Receptor; PR: Progesterone Receptor

## Competing interests

The authors declare that they have no competing interests.

## Authors' contributions

BN carried out the immunohistochemical analysis, performed the statistical analysis, and drafted the manuscript. NZ and EN carried out the immunohistochemical analysis and drafted the manuscript. JB collected the clinical data. JM participated in collection of clinical data and drafted the manuscript. DB performed the statistical analysis and drafted the manuscript. KJ participated in the conception and design of the study, performed the histopathological re-evaluaton and drafted the manuscript. All authors read and approved the final manuscript.

## References

[B1] Jemal A Siegel R Ward E Hao Y Xu J Thun MJ Cancer statistics, 2009 CA Cancer J Clin 2009 59 4 225 49 10.3322/caac.20006 19474385

[B2] Helzlsouer KJ Alberg AJ Gordon GB Longcope C Bush TL Hoffman SC Comstock GW Serum gonadotropins and steroid hormones and the development of ovarian cancer Jama 1995 274 1926 1930 10.1001/jama.274.24.1926 8568986

[B3] Silva EG Tornos C Fritsche HA Jr el-Naggar A Gray K Ordonez NG Luna M Gershenson D The induction of benign epithelial neoplasms of the ovaries of guinea pigs by testosterone stimulation: a potential animal model Mod Pathol 1997 10 879 883 9310950

[B4] Ludwig A Murawska M Panek G Timorek A Kupryjanczyk J Androgen, progesterone and FSH receptor polymorphisms and ovarian cancer risk and outcome Endocr Relat Cancer 2009 19458022 10.1677/ERC-08-0135

[B5] Schildkraut JM Murphy SK Palmieri RT Iversen E Moorman PG Huang Z Halabi S Calingaert B Gusberg A Marks JR Berchuck A Trinucleotide repeat polymorphisms in the androgen receptor gene and risk of ovarian cancer Cancer Epidemiol Biomarkers Prev 2007 16 473 480 10.1158/1055-9965.EPI-06-0868 17372242

[B6] Cardillo MR Petrangeli E Aliotta N Salvatori L Ravenna L Chang C Castagna G Androgen receptors in ovarian tumors: correlation with oestrogen and progesterone receptors in an immunohistochemical and semiquantitative image analysis study J Exp Clin Cancer Res 1998 17 231 237 9700586

[B7] Chadha S Rao BR Slotman BJ van Vroonhoven CC Kwast TH van der An immunohistochemical evaluation of androgen and progesterone receptors in ovarian tumors Hum Pathol 1993 24 90 95 10.1016/0046-8177(93)90067-Q 8418017

[B8] Lee P Rosen DG Zhu C Silva EG Liu J Expression of progesterone receptor is a favorable prognostic marker in ovarian cancer Gynecol Oncol 2005 96 671 677 10.1016/j.ygyno.2004.11.010 15721410

[B9] Lau KM Mok SC Ho SM Expression of human estrogen receptor-alpha and -beta, progesterone receptor, and androgen receptor mRNA in normal and malignant ovarian epithelial cells Proc Natl Acad Sci USA 1999 96 5722 5727 10.1073/pnas.96.10.5722 10318951 PMC21927

[B10] Dubeau L The cell of origin of ovarian epithelial tumours Lancet Oncol 2008 9 1191 1197 10.1016/S1470-2045(08)70308-5 19038766 PMC4176875

[B11] Berglund G Elmstahl S Janzon L Larsson SA The Malmo Diet and Cancer Study. Design and feasibility J Intern Med 1993 233 45 51 10.1111/j.1365-2796.1993.tb00647.x 8429286

[B12] Berglund G Eriksson KF Israelsson B Kjellstrom T Lindgarde F Mattiasson I Nilsson JA Stavenow L Cardiovascular risk groups and mortality in an urban swedish male population: the Malmo Preventive Project J Intern Med 1996 239 489 497 10.1046/j.1365-2796.1996.483819000.x 8656142

[B13] McShane LM Altman DG Sauerbrei W Taube SE Gion M Clark GM REporting recommendations for tumour MARKer prognostic studies (REMARK) Eur J Cancer 2005 41 1690 1696 10.1016/j.ejca.2005.03.032 16043346

[B14] Kononen J Bubendorf L Kallioniemi A Barlund M Schraml P Leighton S Torhorst J Mihatsch MJ Sauter G Kallioniemi OP Tissue microarrays for high-throughput molecular profiling of tumor specimens Nat Med 1998 4 844 847 10.1038/nm0798-844 9662379

[B15] Kobel M Kalloger SE Boyd N McKinney S Mehl E Palmer C Leung S Bowen NJ Ionescu DN Rajput A Ovarian carcinoma subtypes are different diseases implications for biomarker studies PLoS Med 2008 5 e232 10.1371/journal.pmed.0050232 19053170 PMC2592352

[B16] Edmondson RJ Monaghan JM Davies BR The human ovarian surface epithelium is an androgen responsive tissue Br J Cancer 2002 86 879 885 10.1038/sj.bjc.6600154 11953818 PMC2364138

[B17] Carlson JW Miron A Jarboe EA Parast MM Hirsch MS Lee Y Muto MG Kindelberger D Crum CP Serous tubal intraepithelial carcinoma: its potential role in primary peritoneal serous carcinoma and serous cancer prevention J Clin Oncol 2008 26 4160 4165 10.1200/JCO.2008.16.4814 18757330 PMC2654373

[B18] Lee Y Miron A Drapkin R Nucci MR Medeiros F Saleemuddin A Garber J Birch C Mou H Gordon RW A candidate precursor to serous carcinoma that originates in the distal fallopian tube J Pathol 2007 211 26 35 10.1002/path.2091 17117391

